# Latent Variable Machine Learning Framework for Catalysis: General Models, Transfer Learning, and Interpretability

**DOI:** 10.1021/jacsau.3c00419

**Published:** 2023-12-19

**Authors:** Gbolade O. Kayode, Matthew M. Montemore

**Affiliations:** Department of Chemical and Biomolecular Engineering, Tulane University, New Orleans, Louisiana 70118, United States

**Keywords:** transfer learning, catalysis, latent variable, alloys, material screening

## Abstract

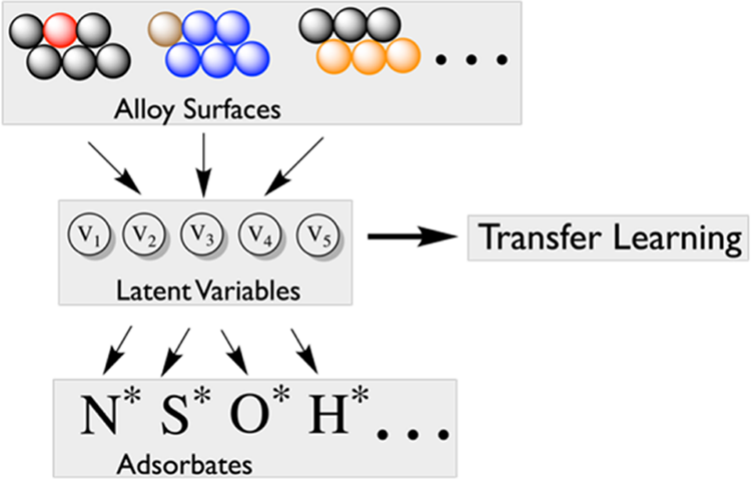

Machine learning has been successfully applied in recent years to screen materials for a variety of applications. However, despite recent advances, most screening-based machine learning approaches are limited in generality and transferability, requiring new models to be created from scratch for each new application. This is particularly apparent in catalysis, where there are many possible intermediates and transition states of interest in addition to a large number of potential catalytic materials. In this work, we developed a new machine learning framework that is built on chemical principles and allows the creation of general, interpretable, reusable models. Our new architecture uses latent variables to create a set of submodels that each take on a relatively simple learning task, leading to higher data efficiency and promoting transfer learning. This architecture infuses fundamental chemical principles, such as the existence of elements as discrete entities. We show that this architecture allows for the creation of models that can be reused for many different applications, providing significant improvements in efficiency and convenience. For example, our architecture allows simultaneous prediction of adsorption energies for many adsorbates on a broad array of alloy surfaces with mean absolute errors (MAEs) around 0.20–0.25 eV. The integration of latent variables provides physical interpretability, as predictions can be explained in terms of the learned chemical environment as represented by the latent space. Further, these latent variables also serve as new feature representations, allowing efficient transfer learning. For example, new models with useful levels of accuracy can be created with less than 10 data points, including transfer learning to an experimental data set with an MAE less than 0.15 eV. Lastly, we show that our new machine learning architecture is general and robust enough to handle heterogeneous and multifidelity data sets, allowing researchers to leverage existing data sets to speed up screening using their own computational setup.

## 1 Introduction

Machine learning (ML) has seen wide applications in screening of molecules and materials^1−6^ and has seen some notable successes in discovering new materials.^7−10^ When applying ML to systems with guest–host chemical bonding, such as catalysis, sorption, intercalation, etc., a separate model is usually created for each guest. For example, applications to catalysis have most often focused on predictions of a single species.^11−14^ This allows accelerated design for a given reaction, and several of these studies have identified promising candidates for high-performing catalysts.^8,14,15^ There have been similar studies using ML for batteries.^2,16^ However, these types of models most often cannot be reused for a new reaction or new battery chemistry; instead, a new model must usually be created from scratch. The need for a new data set and fitting of a new model for each application leads to significant inefficiency. While there has been some progress toward making more general models,^17^ a generalized framework for developing data-efficient, reusable models would greatly improve efficiency for initial screening.

Because ML models used in chemistry and materials science usually predict a single quantity, the design problem is often simplified to allow screening. For example, in catalysis, one or two reaction intermediates are often used as descriptors for the rates along an entire pathway.^18,19^ While this has been quite successful in some cases, it has been shown that this can oversimplify the system in many cases.^20−22^ Because these simple performance descriptors are often developed on a small set of materials that include known high-performance materials, many new materials discovered using ML are similar to materials that were already known to have high performance, despite the large phase spaces that are often searched. ML studies have been criticized for this, and it has been suggested that it may be just as effective for humans to design tweaks to currently known high performers.^23^ For example, ML studies of the electrochemical reduction of carbon dioxide often focus on Cu-based materials,^8,14,15,24,25^ where Cu is well-known to be the best pure metal catalyst. In contrast, predicting many target variables would allow more sophisticated, effective screening on a broad array of materials in addition to providing reusability and increased efficiency.

In nearly all cases, off-the-shelf ML architectures are used to map customized feature sets extracted from the host directly to the quantity of interest. For example, host atoms’ electronegativity, electron affinity, and other similar properties have been used to predict binding energies using random forests, neural networks, linear models, or kernel ridge regression.^11,13−15,26−29^ Graph convolutions, Coulomb matrices, and other similar feature sets have been used to predict atomization energies, formation energies, and binding energies.^11,30−32^

These architectures and feature sets do not incorporate some fundamental properties of chemical systems, such as the fact that chemical systems are made up of discrete elements, whose properties do not vary continuously. Indeed, the chemical space is not continuous, although ML approaches often implicitly assume this. Instead, each element has its own strong chemical identity, which is modified by the environment, and learning the effect of the environment on the bonding propensity for a given element is much easier than learning all of the factors that control the bonding propensity across different elements and environments. Typical ML approaches also do not take advantage of the fact that a few material properties control the binding strength of multiple guest species. For example, frontier orbitals, average energies of electronic bands, and similar electronic properties have been shown to control molecular reactivity,^33^ adsorption energies,^20,34,35^ vacancy energies,^36^ etc. By choosing an architecture that incorporates the properties of chemical systems, we can expect improved data efficiency and generality.

Simple, linear models in catalysis have proven to be powerful tools for understanding and in some cases for screening; however, improving generality, accuracy, and screening efficiency would greatly improve the effectiveness of high-throughput screening, allowing improved prospects for discovering new catalysts. For instance, the d-band model^37,38^ and linear scaling relations^39,40^ are powerful due to their simplicity and interpretability. However, they can suffer from a lack of accuracy and generality, particularly when considering a broad material space.^41^ The lack of accuracy of linear scaling relations on the specific data sets we study here has been noted previously.^20,40,42^ Furthermore, many ML-based studies have predicted energetics on alloys using electronic structure properties;^14,43−48^ however, these studies most often either require density functional theory (DFT)-calculated features, which makes the screening several orders of magnitude slower, or only consider a relatively small range of adsorbates and/or metals, limiting their generality. Additionally, fitting a model to multiple species has been performed in previous work^45,49,50^ but true transfer learning, in which a pretrained model is used to accelerate training for a new application, has rarely been used across adsorbates, across computational setups, or to experimental data.

In other ML fields, such as image recognition, recent advances have obviated the need for fitting a new model from scratch for every application. Generally, pretrained models have been created that are directly applicable to many possible applications and are useful in transfer learning.^51^ For example, ResNet models can classify images into 1000 different classes,^52^ leading to wide applicability and utility for transfer learning.^51^ Similar developments in ML for materials and chemistry would significantly improve the efficiency of materials design, but there are inherent differences between the fields. In the chemical sciences, data are typically more expensive to generate than for image recognition. Hence, typical data sets in materials science are 10^2^ to 10^5^ data points, while other fields that employ deep learning often have data sets with 10^6^ to 10^9^ points. On these smaller data sets, traditional ML approaches are typically more accurate than deep learning.^51^ Further, when using ML for scientific applications, interpretable models are desired, as they can bring new scientific insights. In addition to these challenges, there are some advantages for ML in the chemical sciences. First, chemistry has an underlying structure with fundamental principles, which can be leveraged when building an architecture. Additionally, it is often desirable to either identify candidates for further study or understand broad trends, rather than creating a fully automated system, and hence, extremely high accuracy is often not needed because specific model predictions can be checked prior to more extensive material testing. By adapting strategies from other ML fields (e.g., pretrained models and transfer learning), while accounting for inherent differences between the fields, we can improve the effectiveness of ML for the chemical sciences.

While current ML approaches to predicting adsorption energies can show fairly high accuracy, there is significant room for improvement for a variety of important use cases. For instance, models with high data efficiency but less accuracy (combined with subsequent DFT validation) can overall be much more efficient for initial screening than data-inefficient but highly accurate models. That is, it is more efficient to generate a small training set, screen approximately, and then test a number of promising candidates with DFT, rather than to generate a very large training set and then test only a few promising candidates. Because the generation of very large training sets takes significant resources, reducing the need for these types of training sets is highly desirable. Additionally, most existing frameworks do not provide a clear strategy for leveraging an existing data set to accelerate screening for a different adsorbate species or computational setup, particularly when considering a wide variety of applications and for cases that deviate from linear scaling relations. Furthermore, making predictions for many different species, e.g., all of the species in a reaction pathway on a large number of alloy surfaces, is also quite difficult. Overall, the need for large data sets, and the lack of transferability and generality, are significant weaknesses for most current ML approaches.^49,53^ While previous approaches to transfer learning have shown the ability to transfer across different types of alloy surfaces,^49^ a framework that facilitates transferring across different species, computational parameters, and domains (for example, DFT to experiments) would greatly facilitate the use of existing data sets to accelerate screening.

For this work, we developed a general, interpretable, and transferable latent variable framework for predicting adsorption energies. Our new latent variable framework leverages chemical principles, such as the existence of elements as discrete entities (and not as part of a continuous space) in its learning process. We demonstrate the effectiveness of this new framework in transfer learning across chemical species, across computational setups, and from computational data to experimental data. Furthermore, we showcase the framework’s utility in representation learning, as new feature representations can be learned in the form of latent variables based on the input features. These latent variables correlate with the well-established d-band center, supporting the inherent interpretability of our framework. Lastly, we further show that our framework is robust enough to handle heterogeneous and multifidelity data sets.

## 2 Methods

The adsorption energy data sets used in this work were taken from the Montemore^54^ (expanded somewhat beyond what was used in previous work) and Mamun^42^ data sets. Although these data sets are distinct in sizes and computational setups, they both contain adsorption energies of a variety of adsorbates on bimetallic alloys. Montemore’s data set (smaller) was generated from DFT calculations using the PW91 functional^55^ and the projector augmented wave method^56,57^ using the VASP software.^56,58^ These calculations were performed for C, N, O, OH, H, S, K, F, CH_3_, CH_2_, OH, and CH_3_O on surface alloys generated from the combination of 23 transition metals. These metals include Cu, Ag, Au, Ni, Pt, Pd, Co, Rh, Ir, Fe, Ru, Os, Mn, Re, Cr, Mo, W, V, Ta, Ti, Zr, Hf, and Sc. Individual adsorbates were placed in a 3 × 3 supercell containing 4 layers of the alloy surface with the bottom two layers fixed and the top two layers allowed to relax. In the end, a total of 1321 adsorption energies were generated. For Mamun’s data set (larger), the BEEF–vdw functional^59^ and Quantum Espresso software^60^ were used to perform DFT calculations. In this case, the adsorbates were placed in a supercell containing 3 layers of alloy with the bottom two layers fixed and the top layer allowed to relax. Although this data set contained intermetallic alloys from a combination of 37 metals, we only selected combinations from the same 23 metals present in the Montemore data set to keep both sets consistent. The adsorbate species we selected were H, N, C, O, CH_3_, CH_2_, CH, and NH, yielding a total of 13964 adsorption energies. Mamun’s data set is about ten times larger than Montemore’s data set; thus, we denote these sets as the “larger data set” and “smaller data set” respectively. It is important to note that the two data sets are not directly comparable due to differences in computational methods, functionals, alloy configuration, and unit cell sizes for energy calculations. Such differences are often encountered in the field, as researchers often have access to multiple data sets generated under different conditions and assumptions. We also leveraged CO experimental data from previous work.^5^ This data set is composed of experimentally determined CO adsorption energies on pure metals. Furthermore, the density of states (DOS) data used in this work for computing the d-band centers was taken from previous work.^54^ This DOS data set consists of 211 metal surfaces (mostly alloys with a few pure metals) calculated using a single-point calculation on the relaxed metal geometry for a total of 1213 unique data points. These metal surfaces were constructed from a pool of 16 elements: Ni, Rh, Ir, Ru, Os, Co, Re, Ag, Au, Cu, Sc, Hf, Zr, Ti, Pt, and Pd.

## 3 Results and Discussion

### 3.1 Model Architecture

Our goal is to develop a model architecture that allows efficient learning of the adsorption energies of many species. To do this, we leverage our domain knowledge in chemistry and materials science in two ways. First, many previous studies^41,46,61−67^ have established relationships between the properties of a material or molecule and its propensity for bonding with different species. For example, the frontier orbitals,^66,67^ d-band center,^34,64,65^ electronegativity,^41,68^ reduction potential,^63^ and similar properties have been shown to control or influence bonding in various materials and influence bonding to multiple species. Once these material properties are known, we do not need any specific information about the host identity. The existence of these material properties suggests the use of latent variables to stand in for physical properties and predict a material’s affinity for multiple species. This allows us to decouple the host material from the guest species, which greatly reduces the combinatorial complexity of considering many guests and hosts. Second, materials are made up of a relatively small number of elements, each of which has its own unique identity that is perturbed by the local environment. Therefore, we can fit a separate model for each host element to predict how the local environment changes the latent variables and ultimately the bond strengths.

In statistical learning, latent variables are intrinsic quantities that cannot be directly measured but link observable variables.^69−71^ Many physical systems are characterized by these “hidden” features. In our case, the latent variables will replace physical material properties that control bonding but are not trivial to calculate, such as electronic structure properties. These will link the observable variables, which will be adsorption energies (our target variables) and material properties that are easy to extract from the structure (our features). Using latent variables rather than explicit material properties improves accuracy, makes data collection simpler, and removes the need for a priori identification of which material properties affect binding strength.

Latent variables have been used to simplify learning processes or create interpretable models in many fields; here, we use them primarily to simplify learning when we have multiple target variables.^69,71−74^ We hypothesized that replacing a complex, all-encompassing, and opaque model with an ensemble of simpler models would greatly simplify the entire learning process based on the physical insights noted above. More generally, the right combination of weak learners has been proven to be quite effective, as shown by boosted decision trees.^75^ Furthermore, latent variables also help capture interactions between observable quantities, as shown in factor analysis.^76^ Oftentimes, there are dependencies between observable quantities, and the use of latent variables helps incorporate that into the model. In our case, it is known that there are often relationships between adsorption energies of different species, but these relationships are not always simple.^19,46^ Using latent variables allows us to leverage these relationships without making assumptions about their form.

**Figure 1:**
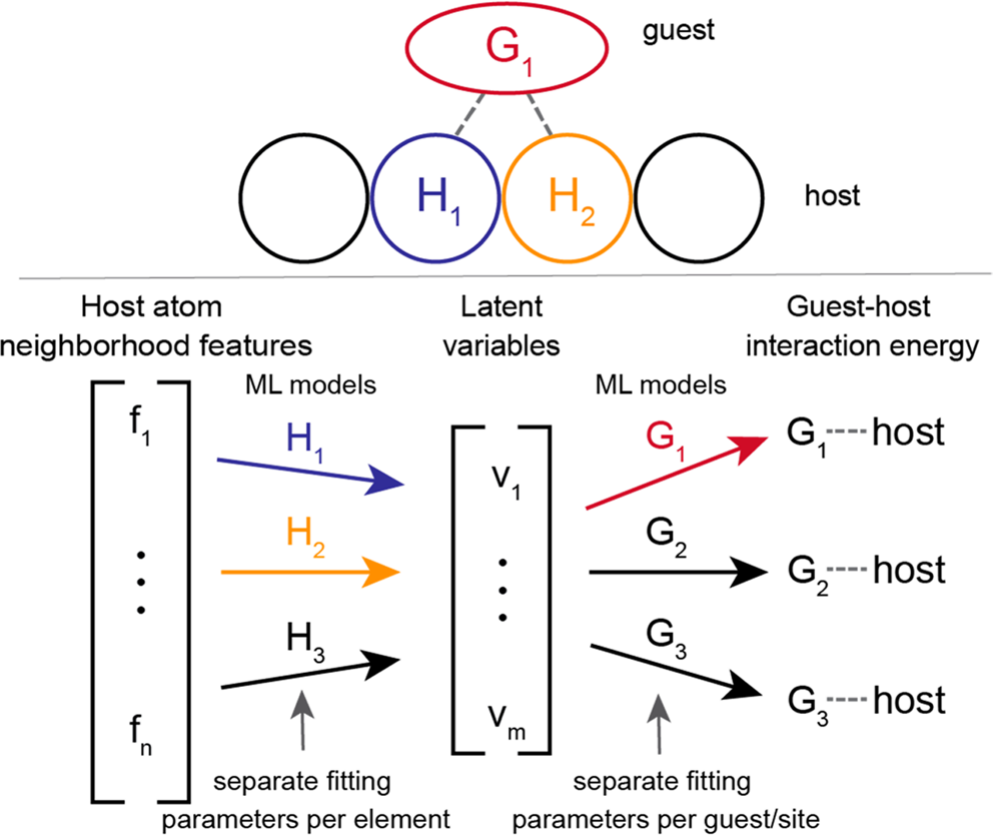
Latent variable ML architecture applied to a simple guest (adsorbate) bound to a host (potential catalyst). Here, the guest G_1_ is bound to a mixed site of the two different elements H_1_ and H_2_. All submodels are fit simultaneously, such that the latent variables are implicitly determined by the model.

To more concretely demonstrate our latent variable architecture, consider a generic catalytic system with a guest molecule or atom (G_1_) adsorbed to a mixed site (H_1_ and H_2_) on the surface or within a host material (e.g., a bridge site on an alloy surface containing two different elements). The host material is a potential catalyst in our case (Figure 1), but more generally, it could be a sorbent or battery material. In order to predict the effectiveness of the catalyst, it is important to know the strength of interaction between the guest and host. Here, rather than directly mapping the observable quantities (host atom neighborhood features) to the target (guest–host interaction energies), we use latent variables to decouple the host from the guest and site. This substantially decreases the complexity associated with having multiple guests and hosts. The decoupling allows us to fit a different model for each host element *H* to describe how its atomic environment affects a latent variable *v*. This is represented by the first set of submodels in Figure 1. Likewise, we are also able to fit a different model for each guest *G* and site to describe how the guest–host interaction energies depend on the latent variables *v*. This is represented by the second set of submodels in Figure 1. All submodels are fit simultaneously, such that the latent variables are implicitly determined by the framework. This means that the framework will determine latent variable values that most accurately map to the set of guest–host interaction energies. Furthermore, the use of latent variables that decouple the guest from the host allows predictions for specific guest–host combinations that are not in the data set, as long as the guest and host are separately present in the data set. In this framework, each submodel is given a relatively simple learning task, in contrast to traditional descriptor-based methods^77^ that take on a complex learning task all together using features characterizing the host, and sometimes guest. Thus, rather than developing complex descriptors (for example, the SISSO method^77^) and/or complex models for predicting a broad set of adsorption energies, our framework uses submodels connected by latent variables to transform the learning task in a way that is particularly suited for broad data sets (see Sections S1 and S2 for more information on the mathematical description as well as a detailed example of our framework.)

In principle, our architecture offers the flexibility of choosing any model type for the submodels. The guest submodels are required to be linear in our current implementation, while the host submodels can be linear or nonlinear. The linear approach is highly interpretable and enables easy extraction of the latent variables and all fitting parameters. By enabling the extraction of latent variables, our framework is analogous to autoencoders, where it serves as a generative model for new feature representations based on the input features. This is known as representation learning and we discuss it further in Section 3.3. Additionally, the linear approach has high bias and low variance and is therefore less prone to overfitting.

We tested several nonlinear host submodels and found that the extreme gradient boosting (XGBoost)^78^ regressor gave the most accurate predictions when compared with other nonlinear methods. Using nonlinear host submodels can be more accurate than using linear submodels, particularly when considering a large data set. Although this approach can give a more accurate model, it is less interpretable and makes it difficult to extract the latent variables. Therefore, there are trade-offs between using linear and nonlinear submodels. The linear version has clear advantages in interpretability and latent feature generation, while the nonlinear version often gives higher predictive accuracy. Linear models are also very fast at making predictions, which is useful when screening a very large number of materials. We applied both the linear and nonlinear versions of our framework in this work to elucidate the advantages of each.

For the host neighborhood feature set, which are the inputs to our framework, we primarily leveraged host features used in previous work.^54^ These features were originally selected by using a combination of data-driven techniques and physical intuition to determine the best feature set from a large pool of features. This feature selection technique balances accuracy, interpretability, and mitigation of researcher bias. More details and the physical justification for these features can be found in this previous work.^54^ The selected features for describing the environment of a given atom are the d-band filling, the bulk electrical conductivity of neighboring atoms, and the p–d coupling between an atom and its neighbors. The d-band filling of neighboring atoms appears in two variations in this previous work,^54^ and we preserve this structure in the current work. Specifically, one variation only has the d-band filling of the neighboring atoms, while the other has the d-band filling of the central atom multiplied by the neighboring atoms. In essence, the feature set consists of four variables that are made from three distinct features. We found this feature set to give good accuracy in the current work and it was used throughout this work, unless otherwise specified. We tested a feature set with four distinct features, p–d coupling between an atom and its neighbors, electrical conductivity of the neighboring atoms, electron affinity of the neighboring atoms, and the group number of the neighboring atoms and found that this can improve accuracy as described below, although the higher complexity will somewhat lessen the interpretability. In general, standard feature selection techniques can be used with our framework to identify an accurate feature set. We also note that none of these features require any DFT calculations, and they are either tabulated or easily calculated based on the geometry of the unrelaxed structure of the host.

### 3.2 Model Accuracy and Data Efficiency

**Figure 2:**
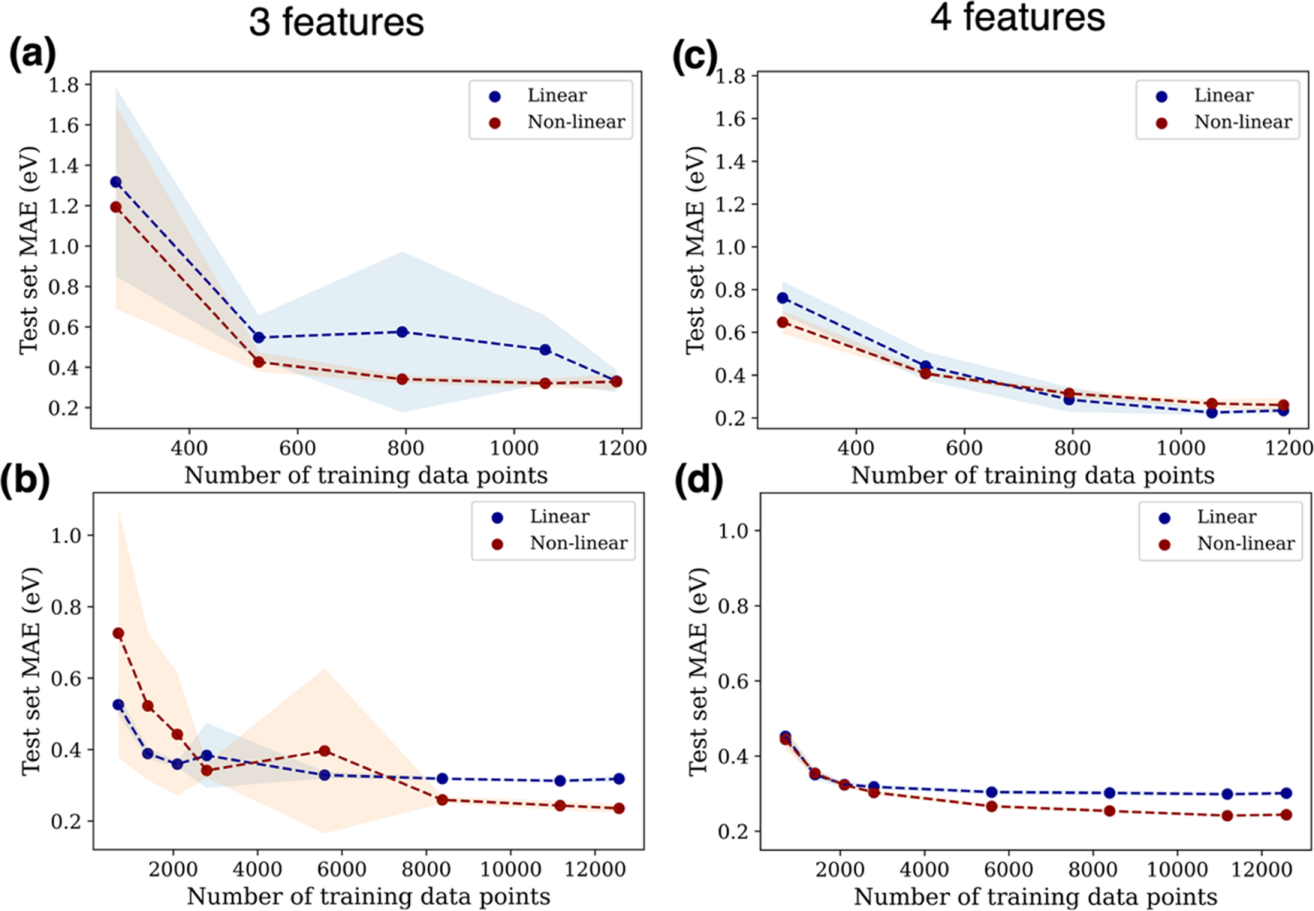
Learning curves (errors, with standard deviations shaded vs training set size) for linear and nonlinear versions of our latent variable architecture constructed with (a, b) 3 features or (c, d) 4 features and applied to (a, c) the smaller data set, which contains adsorption energies on surface alloys, and (b, d) the larger data set, which contains adsorption energies on bulk alloy surfaces.

In Figure 2, we show the learning curves for our latent variable framework applied separately to the smaller and larger adsorption energy data sets using 3 and 4 features (see previous section for description of feature sets). The mean absolute error (MAE) and standard deviation on the test sets were calculated for varying amounts of training data using a standard cross-validation technique repeated 5 times. When using three features, both versions of our framework give reasonably accurate predictions, with the linear and nonlinear version attaining MAEs of 0.33 ± 0.05 and 0.33 ± 0.04 eV for the smaller data set (Figure 2a) and 0.32 ± 0.01 and 0.24 ± 0.01 for the larger data set (Figure 2b) at 90% of the data set. For the smaller data set, the nonlinear version is more accurate, although both versions have fairly similar errors in most cases and the standard deviations overlap. For the larger data set, the nonlinear version is more accurate for larger training data set sizes but less accurate for smaller data sizes. Thus, for the larger data set, when using only a small training set, using the simpler linear submodels gives higher accuracy, but when using a larger training set, the higher flexibility of the nonlinear submodels gives higher accuracy.

Various use cases involve trade-offs between accuracy, data efficiency, interpretability, etc. To improve accuracy, in particular, we repeated the process above but with a somewhat larger feature set. The new feature set includes sd-coupling, electrical conductivity, electron affinity, and the group number. This new feature set was identified by testing various 4 feature combinations. The resulting learning curve is shown in Figure 2c,d. On the small data set, the linear and nonlinear models attain an MAE of 0.22 ± 0.01 and 0.26 ± 0.03 eV, respectively. Comparing this with the previous 0.33 ± 0.05 and 0.33 ± 0.04 eV, this showcases a significant decrease in error of up to 30%. For the large data set, the linear and nonlinear models attain an MAE of 0.30 ± 0.004 and 0.24 ± 0.004 eV, respectively, compared with the previous 0.32 ± 0.01 and 0.24 ± 0.01. This represents a 6% decrease in error along with generally narrower confidence intervals. For this larger data set, the linear version performs reasonably well with training set sizes of less than 2000 data points, above which the nonlinear version significantly outperforms it. Overall, our framework achieves an MAE of 0.22 and 0.24 eV on the small and large data sets, respectively, with these 4 features, even with a large number of adsorbates and metals present.

In general, the good accuracy and data efficiency shown on these very heterogeneous data sets containing many adsorbates and metals demonstrate the utility of our latent variable framework (Figure 2). For example, with roughly 10^3^ data points, we obtain models with useful levels of accuracy on data sets that include roughly 10 adsorbates on alloys of dozens of different elements. As these models could be applied to >10^8^ different adsorbate–surface site combinations, 10^3^ data points represent a very small portion of the phase space. The good accuracy at modest data set sizes indicates that our framework transforms the learning problem in a useful way, such that learning is efficient. This is further corroborated by the fact that linear submodels also attain reasonably high accuracy, as this suggests that our framework significantly reduces the complexity of the learning task for each submodel.

### 3.3 Transfer Learning

**Figure 3:**
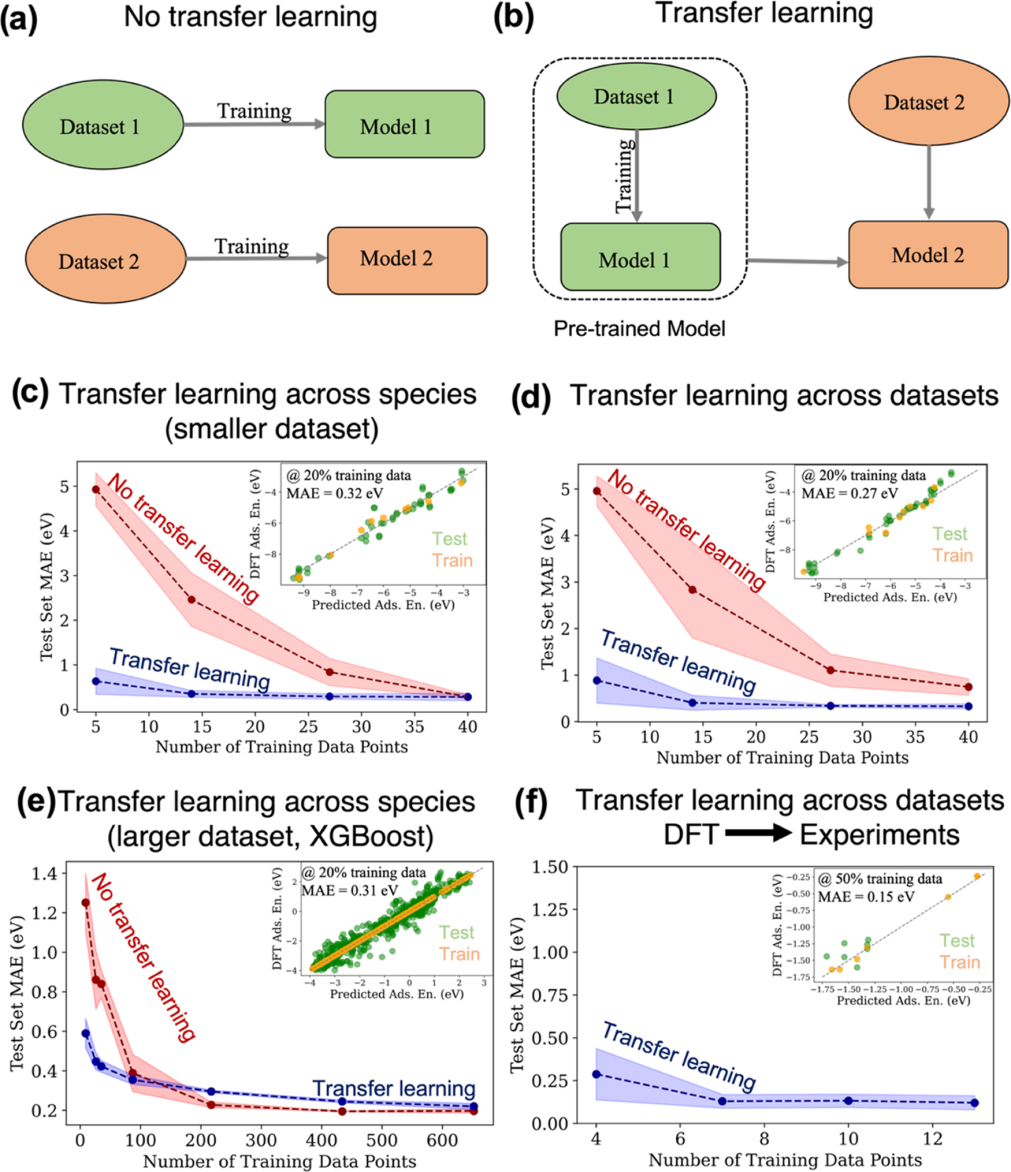
(a) Conventional ML approach, where each model is created from scratch for the different domains represented by each data set. (b) Transfer learning approach: instead of creating each model from scratch, we leverage prior knowledge acquired from a previous learning task via a pretrained model. This can accelerate the learning process. (c) Learning curves for transfer learning across species with the smaller data set. O is completely left out of the pretrained model, which is then used to accelerate learning for O by fitting a linear model to the latent variables. (d) Learning curves for transfer learning across data sets. A pretrained model was fit to the larger data set and then used to accelerate learning on O adsorption data from the smaller data set with a linear model. (e) Transfer learning across species (to O) with the larger data set, using XGBoost to predict the adsorption energies from the latent variables. (f) Transfer learning from DFT data to experimental data. A pretrained model was fit to the larger DFT data set and then used to accelerate learning on experimentally determined CO binding energies using a linear model. All pretrained models used linear submodels.

Traditional ML techniques are devised to learn in isolation with each model built from scratch for different specific applications. However, the concept of transfer learning breaks this paradigm by leveraging prior knowledge acquired from a previous learning task or a pretrained model. Consider data sets 1 and 2 (Figure 3a,b) from two different domains. Without transfer learning (Figure 3a), the learning tasks are performed independently of each other. In contrast, for the transfer learning case (Figure 3b), knowledge gained by fitting a model to data set 1 (the source domain) is used to improve learning efficiency and/or accuracy when fitting a model to data set 2 (the target domain). Often, data set 2 is smaller than data set 1, and it is desired to leverage data set 1 to create a more accurate model for data set 2 than would otherwise be possible for a small data set. Overall, transfer learning can enable the creation of accurate, data-efficient models for new applications by leveraging existing data and models.

Our framework is particularly useful for transfer learning because it uses latent variables to learn and accumulate knowledge, which makes the knowledge easy to access and transfer across domains. Specifically, during training, the framework identifies a latent variable representation that is a transformation of the input variables and is broadly useful for predicting many related output variables (i.e., adsorption energies of several species). This latent representation is likely to also be useful for predicting additional related output variables. Thus, our framework is suitable for transfer learning because it is constructed specifically to build general models, which are likely to contain information that is useful outside the training domain when trained on a robust training data set with multiple guests.

To demonstrate the utility of our framework in transfer learning, we created pretrained models and then applied them to accelerate the creation of predictive models for hollow-site O adsorption energies. After creating the pretrained model, we used its latent variables as input to a new model, which maps from the latent variables to the adsorption energy. This corresponds to keeping the host models and parameters fixed in Figure 1 but fitting a new guest model. This is analogous to techniques used in image recognition, where a new model is sometimes created for a new data set by fitting to the output of hidden layers in a neural network.

First, we tested whether a pretrained model that was fit using data for several species could accelerate learning for a new species. In this case, the source domain consists of all adsorption energies present in the smaller data set except for the O adsorption energies, while the target domain consists of only hollow-site O adsorption energies. We fit our framework to data from the source domain to construct the pretrained model using linear submodels. Next, we fit a linear model from the latent variables of the pretrained model to data from the target domain. In Figure 3c, we show the learning curve for the transfer-learned model together with the no-transfer case, where we fit our framework directly to the data in the target domain from scratch. Both learning curves were created using a standard cross-validation technique repeated 10 times with 10, 25, 50, and 75% of the target domain data set used for training. Transfer learning from the pretrained model is much more data efficient than fitting to the target domain from scratch, as accurate transfer-learned models (MAE = 0.32 eV) can be created with just 10 training points (which is 20% of the data in the target domain as shown in Figure 3c). Furthermore, our framework also shows data efficiency even in the no-transfer case, where the framework is fit to the target domain from scratch, allowing reasonable models to be produced with just 40 data points.

Next, we repeated the same process but used the larger data set as the source domain and O adsorption energies from the smaller data set as the target domain. That is, we pretrained a model using the large data set and used its latent variables to fit models to O adsorption energies from the smaller data set. It is common for different adsorption energy data sets to use different computational parameters, and we thus tested whether an existing data set can be used to accelerate learning for a different set of computational parameters. The learning curves for the no-transfer and transfer-learned models (Figure 3d) again show significantly higher data efficiency when transfer learning is used. Accurate transfer learned models (MAE = 0.27 eV) can be created with just 10 training points (which is 20% of the data in the target domain, as shown in Figure 3d). Further, at 40 data points, the transfer-learned model is significantly more accurate than the no-transfer-learned model. We attribute this improved accuracy to the larger amount of data present in the source domain aiding in determination of the latent variables. Hence, this shows the utility of our framework in transfer learning across data sets with different computational setups, unit cell sizes, and alloy configurations (bulk alloys vs surface alloys). This suggests that using existing data sets can significantly improve the efficiency of ML-based screening when using our framework, even if there are significant differences in computational setup between the existing data set and the target data set.

Next, we tested transfer learning across species using the larger data set. The source domain consisted of the larger data set with all of the O adsorption energies removed, while the target domain consisted of only hollow-site O adsorption energies from the larger data set. Figure S3 shows the learning curve for the linear transfer and no-transfer learned models, which were created using a standard cross-validation technique repeated 10 times for various training set sizes. From Figure S3, the transfer-learned model achieves an MAE of 0.38 eV at 20% of the training data, which are 173 training data points and 696 test data points. The transfer-learned model gives more accurate results up to 100 data points, after which the no-transfer model gives more accurate results. This is one of the limitations of using a linear model to map the latent variables to the target domain: the linearity assumption is not flexible enough to match the accuracy of fitting a complete model from scratch on a larger data set (in this case, >100 data points). This suggests the use of a more sophisticated model for larger target data sets. We then repeated the above process, but instead of transfer learning with a linear model, we used the XGBoost model and again fitted to the latent variables of the pretrained model (Figure 3e). Using XGBoost generally gives higher accuracy than the linear model. Further, the transfer-learned model eventually converges with the no-transfer learned model for larger training sets, which confirms that a more sophisticated nonlinear model is appropriate for transfer learning on larger data sets. Although the no-transfer learned model is still somewhat more accurate for cases greater 100 data points, the transfer learned model is also quite accurate for these cases. Further, at 20% of the training data, the transfer learned model attained an MAE of 0.31 eV and at 75% MAE of 0.19 eV (650 data points).

Lastly, to demonstrate that our transfer-learning framework can indeed be leveraged for experimental applications, we carried out a transfer-learning task using experimentally determined CO binding energies. Our pretrained model was constructed using the same source domain as Figure S3 (DFT adsorption energies, not including CO), while our target domain was experimentally determined CO binding energies. Figure 3f shows the transfer-learned model; fitting from scratch was not feasible in this case because the CO data set only contained few data points per host element. Transfer learning from the pretrained model (using a linear model) gives an accurate model (MAE = 0.15 eV) with just 7 training points (which is 50% of the data in the target domain as shown in Figure 3f). This represents transfer learning from a lower-fidelity domain (DFT) to a higher-fidelity domain (experiments). It also represents transfer learning across species (since CO adsorption energies were not present in the source domain) and across host structures (alloys to pure metals). Further, our framework would likely make adding new metals more efficient, as the model has already learned the mapping from latent variables to adsorption energies. Therefore, only the new mapping from the host environment to the latent variables needs to be fit.

We have thus demonstrated that our framework can be used to create reusable models that are useful for transfer learning. This could significantly speed up the screening of materials by allowing researchers to leverage existing data sets for new applications. In addition to the strategy we used in Figure 3, other strategies are possible, analogous to different strategies that have been used in other machine learning fields to transfer knowledge across domains. Specifically, for our case, if considering a new guest that is quite similar to a guest in the original data set, a simple regression based on predictions for the old guest may allow predictions for the new guest. More generally, a model built from a combination of multiple guest predictions and latent variables will allow predictions, even in cases that are less similar. This strategy is also corroborated by our previous work,^46,54^ where we developed relationships among adsorption energies of many different adsorbates, often based on surface properties. Furthermore, the pretrained model also may be fine-tuned or refit with the new data, or the latent variables and output variables of the pretrained model could also be combined in different ways, with the feature set of the new model determined using *L*_1_ regularization or other standard feature selection techniques. These strategies have trade-offs when considering simplicity, accuracy, data efficiency, and computational time.

### 3.4 Latent Representation

**Figure 4:**
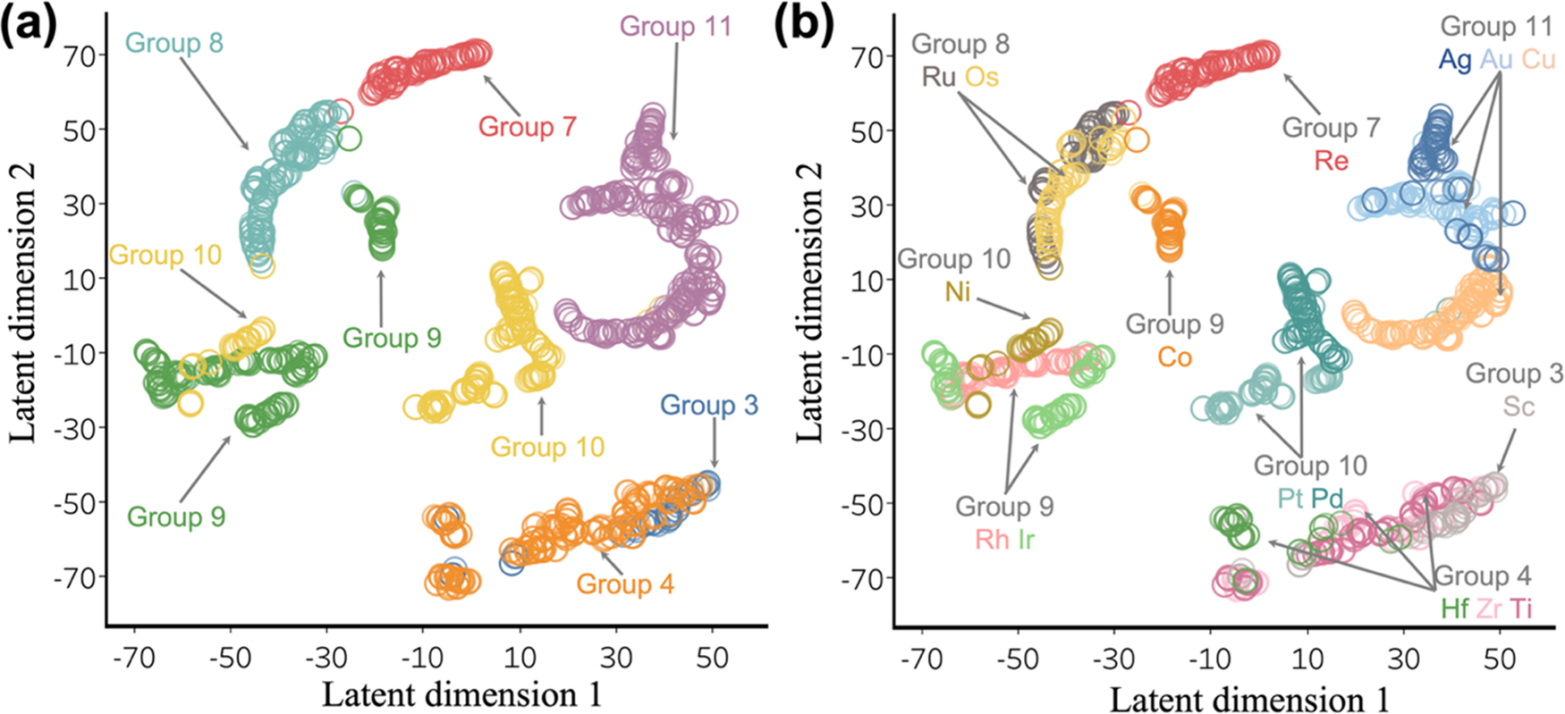
*t*-SNE plots of the latent variable descriptions of all unique surface atoms in the DOS data set. (a) Colored by the group (i.e., column in the periodic table) of each atom. Latent variables within the same group form distinct clusters. (b) Colored by elements. Latent variables belonging to the same element often form distinct clusters within the group clusters.

In the previous section, we leveraged the knowledge gathering capability of our framework by using the latent variables for transfer learning. Our framework’s ability to implicitly generate these latent variables during fitting, which serve as new feature representations for transfer learning, is a form of representation learning. Autoencoders are among the most common algorithms for representation learning, but autoencoders are unsupervised and often need large data sets to be effective. To demonstrate how effective our framework is at learning useful representations that capture a surface atom’s chemical environment, we visualize the latent variables extracted for each unique surface atom in 211 surfaces (DOS data set, 1213 total data points) with the *t*-distributed stochastic neighbor embedding (*t*-SNE) in Figure 4.

We found that latent variables corresponding to elements belonging to the same group in the periodic table are clustered together (Figure 4a). No information about periodicity (either the group or period) was input to the framework. Also, Figure 4b shows that latent variables for a given element in different surfaces often form subclusters within their group’s cluster. For example, the latent variable representations of Ag, Au, and Cu atoms in various local environments each form a cluster, and these clusters form the group 11 cluster. Similarly, the Ru and Os clusters form the group 8 cluster, and the Hf, Zr, and Ti clusters form the group 4 cluster. However, there are a few exceptions: the Ni and Co clusters are not clustered with other elements of their respective groups. This may be because Ni and Co are ferromagnetic and could have different chemical properties compared to elements of their respective groups. This is corroborated by trends in oxophilicity from previous work:^63^ Ni and Co have different oxophilicity compared to the other transition metals in their groups. Further, Ni’s oxophilicity is also quite similar to that of Rh and Ir of group 9; this trend is also seen in Figure 4b, where the Ni cluster is contiguous with the Rh and Ir clusters. In general, we see that our framework learns an important chemical principle, which is that elements not only exist as discrete entities perturbed by their environment but also tend to behave similarly to other elements that are nearby on the periodic table. This further demonstrates the viability of our framework for representation learning.

**Figure 5:**
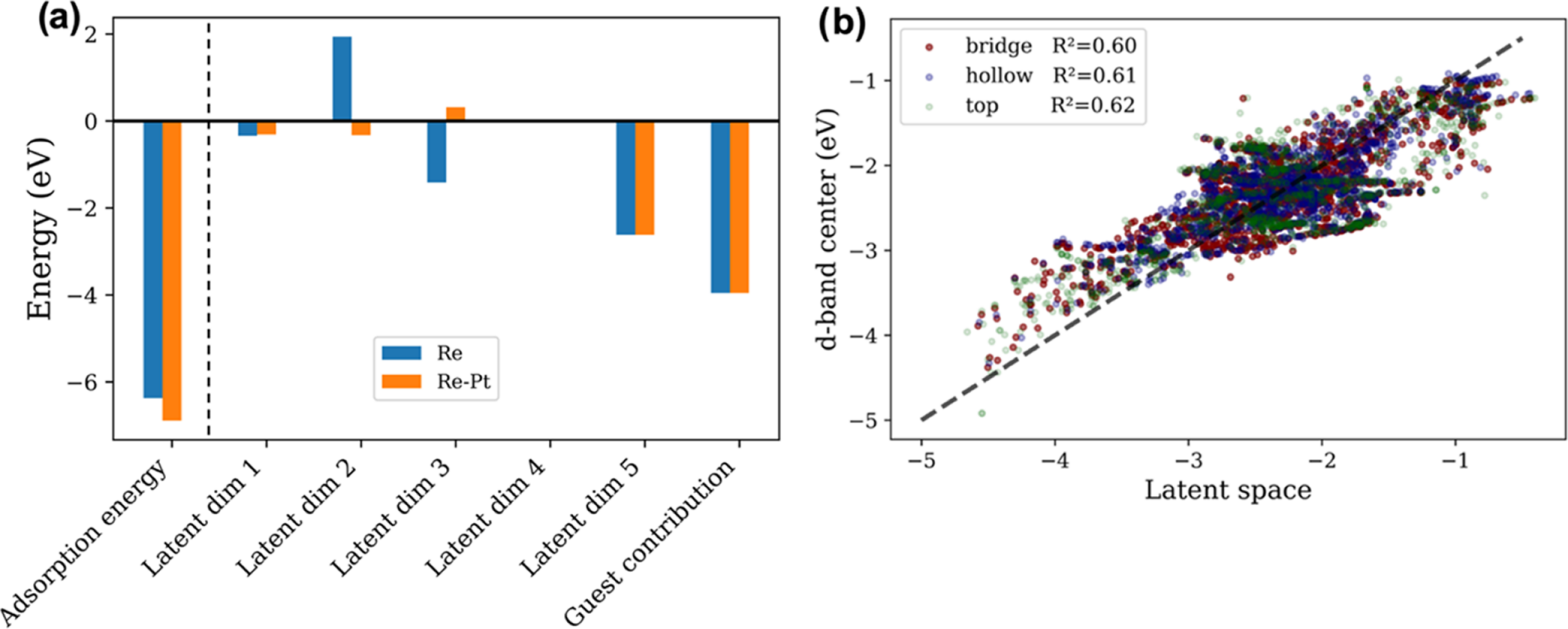
(a) Latent and guest contributions to the adsorption energy of O on Re and Re–Pt. (b) Parity plot showing the linear fit between the latent variables and the d-band center.

Our framework is inherently interpretable when using linear host submodels, as different predictions can be traced quantitatively and directly to changes in host features or latent variables, similar to the energy decompositions in our previous work.^54^ For instance, we can explain why Re–Pt (a Re monolayer on Pt(111)) has a stronger O adsorption energy than Re. Similar trends on Pt-based monolayers have been shown in previous work^79,80^ to affect the activity of the oxygen reduction reaction. For these surfaces, all latent and guest contributions are quite similar or negligible except the contribution from latent dimensions 2 and 3 as shown in Figure 5a. This suggests that the difference in adsorption energies between Re–Pt and Re can indeed be traced back to differences in contributions from these dimensions, which, in turn, can be traced back to the neighbors’ d-band filling and electrical conductivity. This interpretability can be expanded by developing links between latent variables and electronic structure properties. For example, combinations of latent variables can be fit to the electronic structure properties that are calculated for a small subset of materials. A similar strategy has been used in unsupervised learning on X-ray spectra,^81,82^ where latent variables developed from structural and electronic properties were linked to X-ray spectra. To demonstrate this, we fit the latent variables for every unique bridge, hollow, and top site for all metals in the DOS data set to their respective d-band centers. From Figure 5b, we see the latent variables correlate reasonably well with the d-band center with a coefficient of determination (*R*^2^) of 0.60, 0.61, and 0.62 for the bridge, hollow, and top sites, respectively. In this case, we do not expect a perfect correlation as the d-band center alone is not a perfect descriptor of adsorption energy as shown by previous studies.^35,41,83,84^

### 3.5 Heterogeneous and Multifidelity Data

To demonstrate the effectiveness of our framework in handling heterogeneous and multifidelity data, we combined the smaller and larger data set together and fit our framework to the combined data set. This data mixture consists of data from different computational setups, unit cell sizes, and alloy configurations, thereby exhibiting heterogeneity and different levels of fidelity. Separate sets of guest models were fit for each data set, i.e., adsorbates in both data sets had two separate guest models.

**Figure 6:**
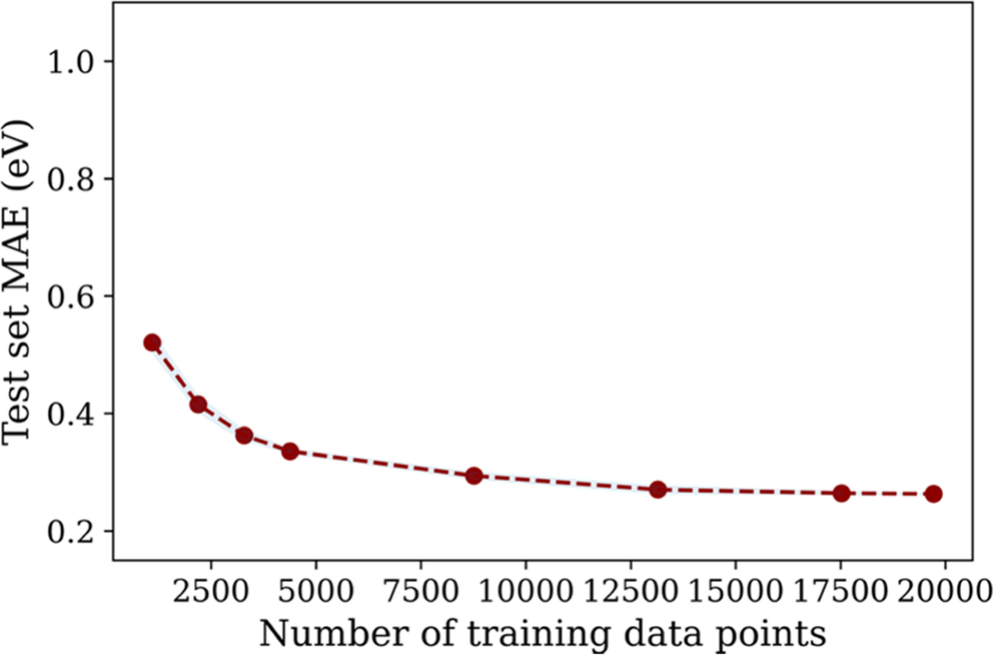
Learning curve (errors, with standard deviations shaded vs training set size) of our latent variable architecture applied to the combined data set (small and large together). The training data was augmented by upsampling the underclass (small data set) due to data imbalance. Ultimately, our framework achieves an MAE of 0.26 eV for the combined data set.

Direct combination of these two data sets would lead to data imbalance since one data set is about 10 times the size of the other. To address this, we upsampled the underclass (small data set) by repeating it 6 times to augment the training data alone and used the 4 features described above during training. We then trained models on varying amounts of the combined training data (with upsampling), with the MAE and standard deviation calculated over 5 random test–train splits. The result is a robust learning curve (Figure 6), which showcases the data efficiency and heterogeneity of our framework at varying training data. Ultimately, our framework achieves an MAE of 0.26 eV for the combined data set and begins to approach this MAE for relatively small fractions of the data set. This shows that our framework is well suited for handling heterogeneous and multifidelity data sets, such as mixtures of various computational and/or experimental data sets.

## 4 Brief Comparison with the Literature

In order to compare our framework’s accuracy relative to previous models, we fit the nonlinear version of our framework from scratch to OH adsorption energies in the larger data set. This subset of data has been used to compare previous models with accuracy ranging from 0.12 to 0.22 eV using a 75%:25% train–test split averaged over 5 independent tests.^85^ We achieved an MAE of 0.21 eV after 5 independent tests, suggesting that our framework is comparable in accuracy to previously published models. Even though our framework had somewhat higher error than the most accurate of the other models, it gives reasonable accuracy while also having simplicity (only 3 input features were used to create accurate models), transferability, a latent representation, interpretability, and the ability to handle heterogeneous and multifidelity data. These attributes highlight the uniqueness of our framework and also its efficiency for material screening.

We also compared both versions of our framework to the widely used GPR model. First, we compared these models using the same three features used in building our models with an additional dummy variable for the site. Figure S2 shows that the latent variable framework is much more data-efficient and accurate than the GPR model using a consistent feature set. Next, we compared the learning curves of our models to the learning curve of a previously published GPR model on the same OH data set.^42^ Our framework is seen to indeed be more data efficient and accurate. For example, we achieve a test set error of around 0.3 eV at 100 data points, as compared to more than 0.4 eV in the previous work. Additionally, we also achieve a test set error of around 0.25 eV at 850 data points, as compared to about 0.35 eV in the previous work.

## 5 Conclusions

In this work, we developed a latent variable framework that is general, interpretable, and transferable and can be reused for different catalytic applications. Our framework leverages chemical principles, such as the existence of elements as discrete quantities. The use of latent variables in our framework also enables it to capture “hidden” and broadly useful knowledge in its learning process and uses a set of submodels, which each take on a relatively simple learning task. This framework allows a single model to efficiently learn across a large number of species and metal alloys and provides clear strategies for transfer learning. We demonstrated the utility of our framework in transfer learning across chemical species and data sets: using a pretrained model built with our framework allows accurate transfer-learned models to be created using just ∼10 data points. We showed the efficacy of our framework in representation learning by generating new feature representations for atoms in different local environments. The generated latent space learned that elements in the same group in the periodic table usually have similar properties. Next, we showcased the interpretability of our framework using energy decompositions and by establishing links between the latent space and a well-known electronic property, the d-band center. Lastly, we show that our framework is robust enough to handle heterogeneous and multifidelity data sets. In general, we have demonstrated that our framework is well-suited for chemical systems and can be used to achieve increased speedup in material screening by allowing reusability. While we focused on predicting adsorption energies that may be useful for heterogeneous catalysis, the same framework can be applied to guest–host interactions more broadly, such as intercalation in batteries or vacancy formation in solid-state materials.

## Data Availability

An implementation of the latent variable models is available at https://bitbucket.org/mmmontemore/surfep.

## Supporting Information Available

Mathematical description of latent variable framework; framework example in predicting O adsorption energy on Re; learning curves of our framework compared with the GPR model; learning curves for transfer learning with linear models across species (to O) with the larger data set; schematic of O adsorbed at the (a) hollow site and (b) top site of a Fe–Ag alloy (PDF)
