# Supplementary material for: Latent Variable Machine Learning Framework for Catalysis: General Models, Transfer Learning, and Interpretability
Source: JACS Au. 2023 Dec 19;4(1):80–91. doi: 10.1021/jacsau.3c00419 (PMC10807004; doi:10.1021/jacsau.3c00419)
Supplement: Supplementary file 1 — au3c00419_si_001.pdf [file au3c00419_si_001.pdf]

Supplementary information for

Latent variable machine learning framework for catalysis: General models, transfer learning, and interpretability

Gbolade O. Kayode[a] and Matthew M. Montemore[a,*]

[a] Department of Chemical and Biomolecular Engineering, Tulane University, New Orleans, LA 70118, USA

*mmontemore@tulane.edu

1 Mathematical description of latent variable framework

Consider a model $p(x)$, where x are observable features. This model could be reformulated by incorporating latent variables v, such that the model becomes $p_g(v)$, where p_g describes how the latent variables are mapped to the observable quantities and $v = p_h(x)$, where p_h describes the behavior of the latent variables. Figure 1 shows this architecture schematically, where $p_g(v)$ corresponds to the second set of models that map the latent variables to adsorption energies, while $p_h(x)$ corresponds to the first set of models that map host atom features to the latent variables. This new architecture could be more accurate depending on the physical system, but more importantly this new architecture can now learn a separate $p_g(v)$, for each observable x, instead of modelling everything with just $p(x)$. We hypothesized that replacing a complex, all-encompassing, and opaque model $p(x)$ with an ensemble of simpler models would greatly simplify the entire learning process, based on the physical insights noted in the main text.

If linear sub-models are chosen, our model equation can be written as:

$$p(x) = \sum_j b_{ij} v_j = \sum_j \sum_k b_{ij} a_{jk} x_k \qquad (1)$$

In contrast, if nonlinear sub-models are chosen, our model equation becomes:

$$p(x) = \sum_j b_{ij} v_j = \sum_j b_{ij} f_j(x_k) \qquad (2)$$

where a_{jk} are the fitting parameters that are different for each host element H and latent variable v_j, and b_{ij} are the fitting parameters that are different for each guest and site. The f_j are host submodels, with a separate model for each host element, while the x_k are the host atom neighborhood features.

2 Framework example in predicting O adsorption energy on Re surface

We show a simple example of our framework in predicting the O adsorption energy on a Re(0001) surface. The model error in this case is 0.08 eV.

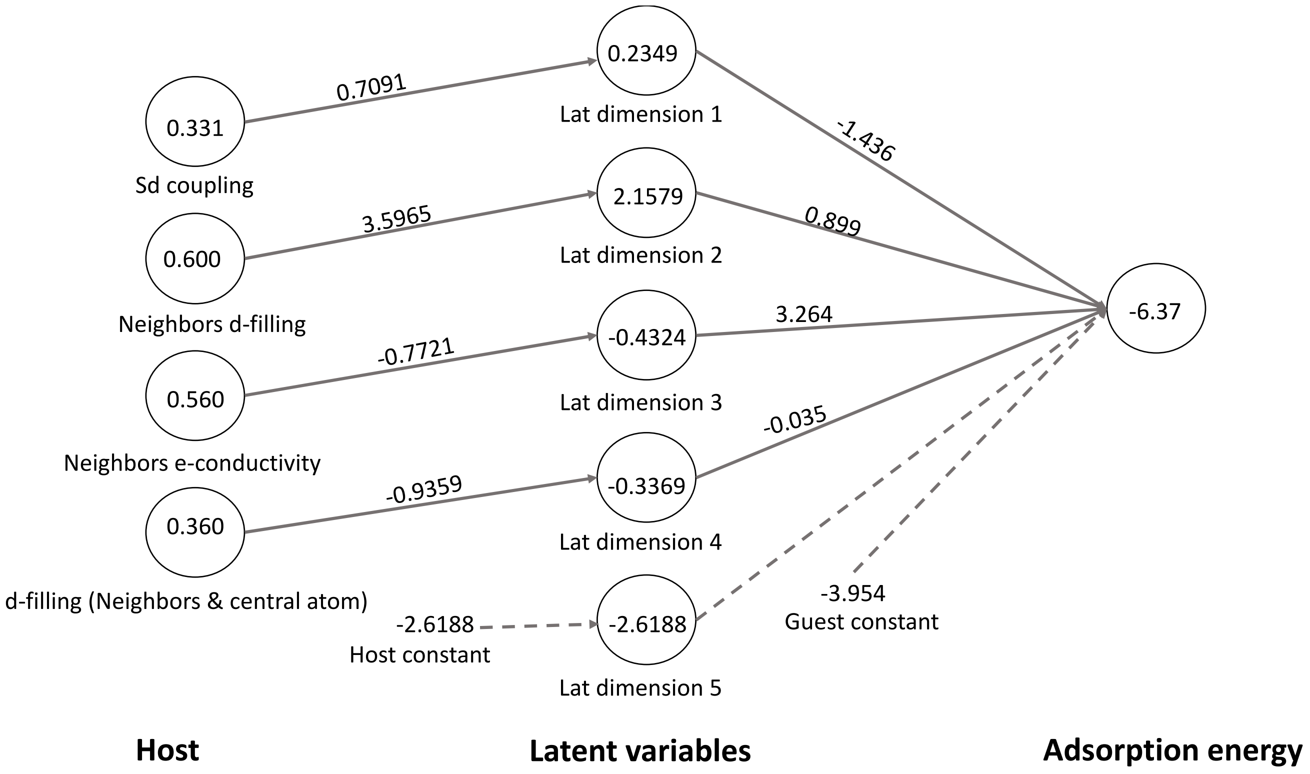

Figure S1: Our framework applied in predicting the O adsorption energy on a Re surface. We show the various energy decompositions involved in our framework.

Table S1: Calculations for the various energy decompositions in Figure S1.

Host		Host weight/bias		Latent variables		Guest weight/bias		Contributions
0.33133	×	0.709071677	=	0.234939555	×	-1.436	=	-0.337373201
0.6	×	3.596467655	=	2.157880593	×	0.899	=	1.939934653
0.56	×	-0.772089576	=	-0.432370162	×	3.264	=	-1.41125621
0.36	×	-0.935879539	=	-0.336916634	×	-0.035	=	0.011792082
Host constant		-2.618830788		-2.618830788				-2.618830788
				Guest constant		-3.954		-3.954
						**Total contribution**		**-6.369733463**

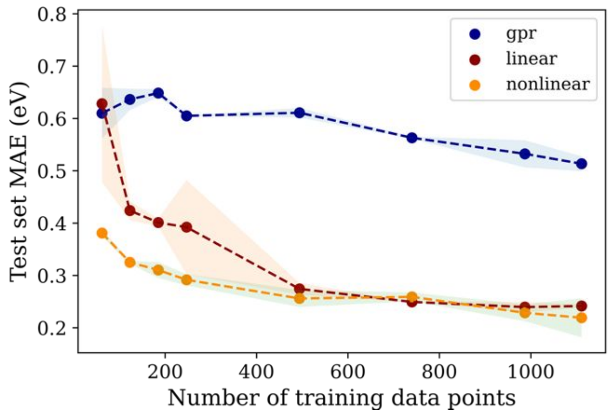

Figure S2: Learning curves for both versions of our latent-variable framework compared with the GPR model using a consistent feature-set but without our framework. We observe that our framework is more data efficient and accurate than the GPR model.

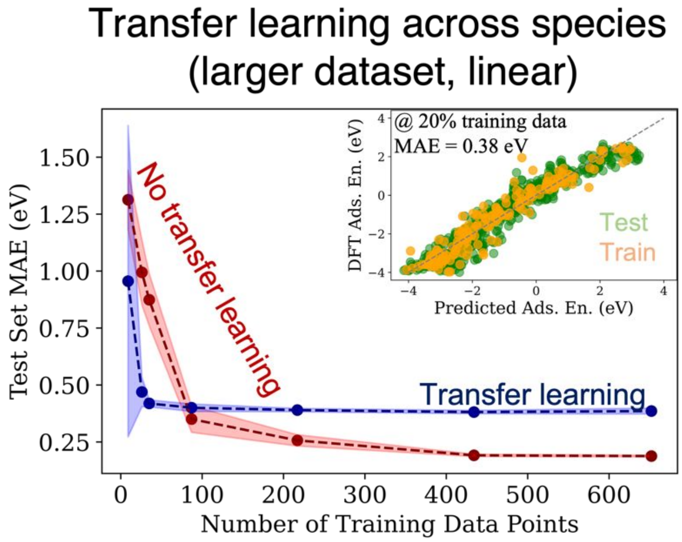

Figure S3: Learning curves for transfer learning with linear models across species (to O) with the larger dataset. Transfer learning is more accurate up to roughly 100 data points, and non-transfer learning is more accurate for larger training sets.

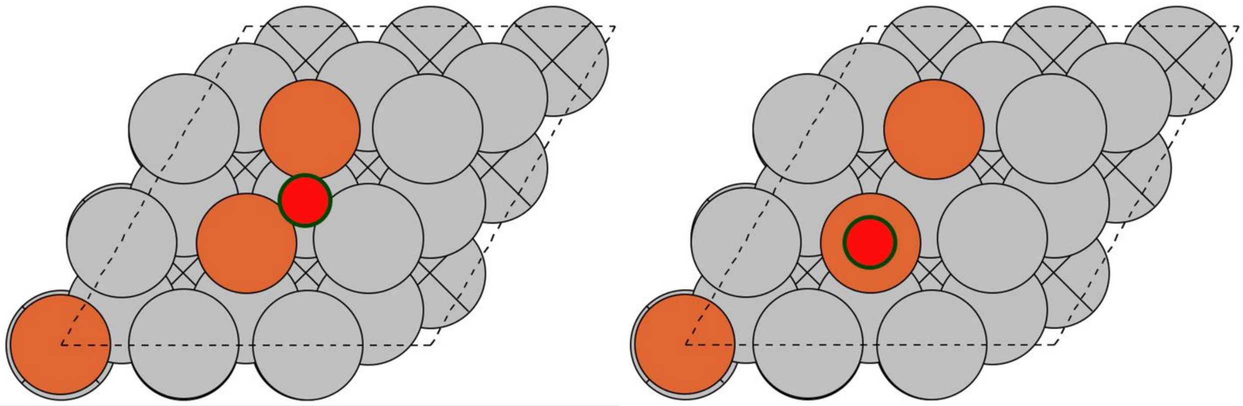

Figure S4: O adsorbed at the (a) hollow site and (b) top site of a Fe-Ag alloy
